# A Quality Improvement Project on Improving Electronic Prescribing System in an Adult Mental Health unit

**DOI:** 10.1192/bjo.2022.346

**Published:** 2022-06-20

**Authors:** Chloe Warner, Matthew Caygill, Suveera Prasad

**Affiliations:** Rotherham, Doncaster and South Humber NHS Foundation Trust, Doncaster, United Kingdom

## Abstract

**Aims:**

Medication tasks are an integral part of a junior doctor's job. However, these can often be timely and use hours that could be spent doing other therapeutic work, especially due to the cumbersome nature of SystmOne. Our aim was to review the amount, type, and time spent on medication tasks and evaluate ways in which the system could be made more efficient and time effective, to release doctors to complete other clinical ward activities.

**Methods:**

We used prospective data collection, with two ten-day cycles carried out across the 46 bedded adult mental health unit (AMHU). Data were collected by all junior doctors working on the AMHU and every medication task was recorded on a designated document at the time of completion. This included data such as time the task was created, related ward, sender, type of task, amount of medications per task and minutes taken to complete.

**Results:**

During the first ten-day cycle of data collection, we found that collectively we spent 21.5 hours completing medication related tasks. 10 hours were spent ordering medications, seven of which were ordering ward stock. Tasks involving completely re-prescribing the medication for the action to be completed took 12.6 hours. The data showed that 42% of tasks were completed on Mondays.

Following cycle one we discussed the data with the AMHU pharmacy team, ward managers and consultants. Subsequent alterations were made to the stocklists for the wards, ward timings were aligned and a collective tasks system created to reduce duplication of tasks.

During the second cycle of data, in total 16 hours were spent on medication tasks. There was a total of 7.45 hours spent ordering medications, 3.35 hours were ordering ward stock. Re-prescribing tasks took 9.7 hours.

**Conclusion:**

From the results of the second cycle of data we can see the recommendations from cycle one have been effective in reducing the amount of time spent ordering medications by 25.8%. This highlights the importance of regularly updating the stocklists and utilising MDT working to maximise efficiency. We also confirmed that Monday was the heaviest day for tasks, which should be considered for staffing. As 46% of overall time was still spent ordering medications, we presented this data at the medicines management committee. Following this, recommendations have been taken to SystmOne for IT system alterations to improve efficiency of the system and allow junior/trainee doctors to focus more time on the clinical care of patients and learning.

